# Mathematical Model-Based Optimization of Trace Metal Dosage in Anaerobic Batch Bioreactors

**DOI:** 10.3390/bioengineering12020117

**Published:** 2025-01-26

**Authors:** Tina Kegl, Balasubramanian Paramasivan, Bikash Chandra Maharaj

**Affiliations:** 1 Faculty of Chemistry and Chemical Engineering, University of Maribor, 2000 Maribor, Slovenia; 2 Department of Biotechnology and Medical Engineering, National Institute of Technology Rourkela, Rourkela 769008, Odisha, India; balap@nitrkl.ac.in

**Keywords:** anaerobic digestion, batch bioreactor, methane production, model parameters calibration, active set optimization method, perturbation of model parameters, gradient based optimization, trace metals

## Abstract

Anaerobic digestion (AD) is a promising and yet a complex waste-to-energy technology. To optimize such a process, precise modeling is essential. Developing complex, mechanistically inspired AD models can result in an overwhelming number of parameters that require calibration. This study presents a novel approach that considers the role of trace metals (Ca, K, Mg, Na, Co, Cr, Cu, Fe, Ni, Pb, and Zn) in the modeling, numerical simulation, and optimization of the AD process in a batch bioreactor. In this context, BioModel is enhanced by incorporating the influence of metal activities on chemical, biochemical, and physicochemical processes. Trace metal-related parameters are also included in the calibration of all model parameters. The model’s reliability is rigorously validated by comparing simulation results with experimental data. The study reveals that perturbations of 5% in model parameter values significantly increase the discrepancy between simulated and experimental results up to threefold. Additionally, the study highlights how precise optimization of metal additives can enhance both the quantity and quality of biogas production. The optimal concentrations of trace metals increased biogas and CH_4_ production by 5.4% and 13.5%, respectively, while H_2_, H_2_S, and NH_3_ decreased by 28.2%, 43.6%, and 42.5%, respectively.

## 1. Introduction

Ever increasing demands for reducing waste and greenhouse gas emissions coupled with increasing renewable energy sources are the driving force for the development of circular waste management technologies such as anaerobic digestion (AD) technology. AD enables reduction of waste entering landfills and has the potential to reduce greenhouse gas (GHG) emissions [1,2,3,4]; the World Biogas Association confirmed in a recent report that AD technology has a potential to reduce GHG emissions in the range between 3290 and 4360 Mt CO_2,eq_, which is equivalent to 10–13% of the world’s current emissions [4]. Furthermore, the production of biogas by AD processes has reached approximately 66 billion m^3^ in 2020, which is equivalent to an energy content of 1.52 EJ. Interestingly, the International Renewable Energy Agency (IRENA) forecasted a tenfold increase in biogas pathways, leading to a supply of 15 EJ by 2050 as part of the Transforming Energy Scenario [5].

The energy, environmental, and financial incentives associated with setting up an AD system necessitates rigorous engineering analysis of the process, where microorganisms in a bioreactor breakdown organic matter (OM) in the absence of oxygen to biogas. The quality and quantity of biogas production depend mainly on AD process conditions, feeding strategy, and feedstock substrate composition (macro and micronutrients). In addition to the microbial community, enzymes and organic degradable matters, such as carbohydrates, proteins, lipids, inorganic compounds, and trace metals (TMs), play a very important role. Although the AD system has been extensively studied, the role of TMs is still under scrutiny and has not sufficiently undergone rigorous engineering analysis to establish tradeoffs between efficiency and cost.

Light metals (Ca, K, Mg, Na, etc.) and heavy metals (Co, Cr, Cu, Fe, Ni, Pb, Zn, etc.) are integral to the biochemical processes occurring in the bioreactor [6,7,8] that facilitate biodegradation. During biodegradation, TMs influence the biogas production quality and quantity by impacting different stages: hydrolysis, acidogenesis, acetogenesis, and methanogenesis. The impact of the TMs on these processes is a consequence of the effect of TMs on microbial consortium activity. Each individual microorganism is affected by various TMs in multiple ways. For example, Co supports the growth and activity of acetogenic bacteria, while Fe, Zn, and Ni are important methanogens building elements [9]. The involvement of TMs (Fe, Ni, Cu, Ca, Mg, and Co) into chemical and physicochemical processes, where sulfide, carbonate, and phosphate precipitates are formed, influences the pH value during the AD process and also the quality and quantity of the produced biogas. Fe can reduce H_2_S in the produced biogas by shifting the chemical equilibrium toward the formation of sulfide precipitates [10,11,12]. Likewise, a Zn concentration below 470 mg L^−1^ can enhance the conversion of organic matter to CH_4_ because zinc ions are a critical element of the active sites in multiple enzymes [13,14]. In general, a lack of TMs reduces microbial growth and activity, which significantly limits the AD process, resulting in accumulation of metabolic intermediates [15]. One intermediate is volatile fatty acids, which cause bioreactor acidification and consequently reduce methane yield. Since some substrates for the AD process may lack adequate amounts of TMs, TM supplementation is necessary in order to maintain an efficient AD process.

Contrarily, many investigations have concluded that an excess of essential TMs may also strongly inhibit the AD process [14,15,16]. It is worth noting that the reported inhibitory concentration of each metal in a bioreactor depends on the interaction of various AD parameters, especially the available microbial community, AD process conditions, and feedstock substrate composition [17]. For example, Chen et al. [18] reported that a concentration of Na over 3.5 g L^−1^ is inhibitory to methanogens at mesophilic temperature, while Geng et al. [19] reported that a Na concentration over 0.35 g L^−1^ can cause hyperosmotic stress, which results in the cellular dehydration of methanogens and a decrease in dehydrogenase activity during food waste AD. Abdel-Shafy and Mansour [20] reported that a Cr concentration over 0.775 g L^−1^ has an inhibitory effect on AD of sludge, while Jha and Schmidt [21] reported that a Cr concentration of 0.012 g L^−1^ may reduce methanogens by 50%. Zhang et al. [16] reported that Fe concentrations over 5.65 g L^−1^ can have inhibitory effects on the AD process of cow dung and Phragmites straw. On the other hand, Wang et al. [22] showed that a concentration of Fe above 2.9 g L^−1^ in the AD of waste-activated sludge can decrease CH_4_ production due to the formation of carbonate precipitates, thereby reducing the ${CO}_{3}^{2-}$ and ${HCO}_{3}^{-}$ content in the bulk phase and decreasing the availability of CO_2_ for hydrogen-utilizing methanogens. Furthermore, Xing et al. [14] investigated the long- and short-term effects of the Zn concentration during excess activated sludge AD and concluded that a Zn concentration over 670 mg L^−1^ reduced methanogenic activities markedly, indicating that an excessive concentration of Zn significantly impairs system stability. These results show that optimal concentrations of metals can vary for various AD scenarios.

As the experiments related to AD are time-consuming, labor intensive, and expensive, numerical simulations (NSs) provide a cost-effective solution when there is a large number of operating parameters with complex interrelationships. So far, NS, which provides a useful tool for AD process understanding and optimization, has been performed using more or less complex mechanistically inspired mathematical models [6,15,23,24,25,26,27,28,29,30,31,32,33,34,35]. It is clear that with the complexity of AD models, which consider various biochemical, chemical, and physicochemical processes, the number of model parameters increases. Since these parameters are unknown or hard to determine, various efficient procedures for model parameters calibration have been developed in multiple studies [28,29,30,35]. However, many researchers calibrate only the most important model parameter, while the estimated values from the literature are typically used for the less important parameters. This simplification neglects the interactions among the less significant model parameters [28,29,30]. Some of these model parameters are related to the activities of TMs, which were included to some extent in some complex AD models [15,26,28,29,30,32,33,34]. For example, Frunzo et al. [15] and Flores-Alsina et al. [26] included the interactions of iron with phosphorus and sulfur into the modeling of the AD process in a CSTR bioreactor. For NS of the AD process in a batch bioreactor, Maharaj et al. [32,33,34] upgraded the AD model by including the activities of Ca, Co, Fe, Mg, and Ni in physicochemical processes; all TM-related model parameters were estimated according to the literature. Kegl et al. [28,29,30] upgraded BioModel for NS of AD performance in a CSTR bioreactor by considering TMs activities; all TM-related parameters were calibrated. However, challenges remain in calibrating and validating such complex models due to the lack of reported data on the range of TM concentrations required for optimal microbial activity, particularly in relation to methane production and other key intermediates in the AD process [34]. Therefore, there is still a need for adequate modeling of the role and activities of all key TMs during the AD process and adequate calibration of all model parameters, including those related to TMs. To the author’s knowledge, no study has yet addressed the importance of accurately calibrating all model parameters. Additionally, there is still a lack of sufficient numerical optimization regarding the optimal concentrations of TMs in the influent feedstock.

This paper investigates the impact of TMs on AD performance, focusing on the modeling, calibration, validation, and numerical optimization of TM concentrations in lab-scale batch bioreactors, with the aim of improving both the understanding and practical application of TM management in AD processes. To explore the impact of various TMs on the AD process, a self-developed BioModel is enhanced to simulate the dynamics of metal interactions throughout the process. The enhanced BioModel incorporates 187 model parameters, all of which require careful calibration. To assess the precision of this calibration, both low and high perturbations of the parameters are thoroughly investigated and analyzed. The calibrated and validated BioModel, tested under diverse operating conditions across different bioreactors, is then integrated into an optimization procedure using a gradient-based approximation method. The optimization aims to determine the optimal quantities of metals added or removed in the influent feedstock, defined using a range of multi-objective and constraint functions. Finally, the results of the BioModel calibration, validation, and numerical optimization are presented and discussed in detail.

## 2. Materials and Methods

At first, the experimental data of the AD process are presented, followed by a detailed description of the enhanced BioModel. In the following, the paper outlines the procedures employed for BioModel calibration and the optimization process, which aims to fine-tune the quantities of trace metals (TMs) in the feedstock to enhance the AD process, using a sophisticated gradient-based approximation method.

### 2.1. Experimental Data

The AD processes of cattle and cow manure in three-batch-mode lab-scale bioreactors (B1, B2, B3) were observed. The bioreactor data are given in Table 1. The animal manure is composed of carbohydrates, proteins, lipids, inert matters, inorganic carbon, inorganic nitrogen, inorganic phosphorus, inorganic potassium, inorganic sulfur in the form of sulfide and sulfate, and other elements and compounds [27,36,37,38]. The input data of the feedstock composition for the AD process in bioreactors B1 [27,36], B2 [37], and B3 [38] are given in Table 2.

### 2.2. Numerical Simulation of the AD Process

To ensure a reliable and efficient NS of AD performance in a batch-mode AD process, an upgraded BioModel was developed and incorporated into the active set optimization (ASO) procedure.

**Enhanced BioModel.** The upgraded BioModel is based on the BioModel presented in [28,29,30], which considers chemical (acid–base reactions), physicochemical (mass transfer from liquid to gas phase and the precipitation), and biochemical (hydrolysis, acidogenesis, acetogenesis, and methanogenesis) processes. In the biochemical reactions, the inhibitory effects of various compounds and TMs on bacteria growth are modeled with non-competitive inhibition.

The main modifications are related to the modeling of the activities of various TMs from the set $I_{TMs}=\left\{Ca,Co,Cu,Cl,Cr,Fe,K,Mg,Na,Ni,Pb,Se,Zn\right\}$ as these TMs are present in the feeding substrate. The effects of all TMs during the AD process are incorporated into the upgraded BioModel using Equations (1)–(8).

At first, the effects of TMs on the pH value in the bioreactor are determined using Equation (1). The $H^{+}$ concentration derivative, needed for pH determination, can be expressed as follows:

(1) $$\frac{d[H^{+}]}{dt}=\frac{d[H^{+}]}{dC_{h}}\;\frac{dC_{h}}{dt}$$

Here, $C_{h}$ denotes the sum of ionic metals concentrations obtained from the following charge balance equation:

$$\begin{array}{ll} C_{h}=\left[{pro}^{-}\right] & +\left[{bu}^{-}\right]+\left[{va}^{-}\right]+\left[{ac}^{-}\right]-\left[{NH}_{4}^{+}\right]+\left[H{CO}_{3}^{-}\right]+2\left[{CO}_{3}^{2-}\right]+\left[H{SO}_{4}^{-}\right]\\ & +2\left[{SO}_{4}^{2-}\right]+\left[{HS}^{-}\right]+2\left[S^{2-}\right]+\left[H_{2}{PO}_{4}^{-}\right]+2\left[{HPO}_{4}^{2-}\right]\\ & +3\left[{PO}_{4}^{3-}\right]+\left[{NO}_{2}^{-}\right]+\left[{An}^{-}\right]-2\left[{Ca}^{2+}\right]-\left[{Cat}^{+}\right]+\left[{Cl}^{-}\right]\\ & -2\left[{Co}^{2+}\right]-2\left[{Cr}^{2+}\right]-2\left[{Cu}^{2+}\right]-2\left[{Fe}^{2+}\right]-\left[K^{+}\right]-2\left[{Mg}^{2+}\right]\\ & -\left[{Na}^{+}\right]-2\left[{Ni}^{2+}\right]-2\left[{Pb}^{2+}\right]-2\left[{Se}^{2+}\right]-2\left[{Zn}^{2+}\right] \end{array}$$

The role of TMs in precipitation is included into the modeling of liquid–solid precipitation rates and the concentrations of precipitates from the set $I_{prec}=\{{CaCO}_{3}$, ${CoCO}_{3}$, ${CuCO}_{3}$, ${FeCO}_{3}$, ${MgCO}_{3}$, ${NiCO}_{3}$, ${PbCO}_{3}$, ${ZnCO}_{3}$, FeS, CoS, CuS, NiS, PbS, ZnS, ${Ca}_{3}{\left({PO}_{4}\right)}_{2}$, ${Co}_{3}{\left({PO}_{4}\right)}_{2}$, ${Fe}_{3}{\left({PO}_{4}\right)}_{2}$, ${Ni}_{3}{\left({PO}_{4}\right)}_{2}$, $Mg{{NH}_{4}PO}_{4}$, $KMg{PO}_{4}\}$. The precipitation rates of sulfides $\rho_{{l-s,S}^{2-}}\;\left(g\;L^{-1}{day}^{-1}\right),$ carbonates $\rho_{l-s{,CO}_{3}^{2-}}\;\left(g\;L^{-1}{day}^{-1}\right),$ phosphates $\rho_{l{-s,PO}_{4}^{3-}}\;\left(g\;L^{-1}\;{day}^{-1}\right)$, and ionic forms of TMs $\rho_{l-s,i}\;\left(g\;L^{-1}\;{day}^{-1}\right),i\in I_{TMs}$ are determined as follows:

(2) $$\rho_{{l-s,S}^{2-}}=\sum_{i}\begin{array}{c} k_{cryst,iS}{\left({\left(c_{i^{2+}}c_{S^{2-}}\right)}^{\frac{1}{2}}-{\left(K_{sp,iS}\right)}^{\frac{1}{2}}\right)}^{2} \end{array},\;\;i\in \left\{Co,Cu,Fe,Ni,Pb,Zn\right\}$$

(3) $$\rho_{l-s{,CO}_{3}^{2-}}=\sum_{i}k_{cryst,i{CO}_{3}}{\left({\left(c_{i^{2+}}c_{{CO}_{3}^{2-}}\right)}^{\frac{1}{2}}-{\left(K_{sp,i{CO}_{3}}\right)}^{\frac{1}{2}}\right)}^{2},\;i\in \left\{Ca,Co,Cu,Fe,Mg,Ni,\;Pb,Zn\right\}$$

(4) $$\begin{array}{ll} \rho_{l{-s,PO}_{4}^{3-}} & =2\sum_{i}k_{cryst,i_{2}{\left({PO}_{4}\right)}_{3}}{\left({\left({\left(c_{i^{2+}}\right)}^{3}{\left(c_{{PO}_{4}^{3-}}\right)}^{2}\right)}^{\frac{1}{5}}-{\left(K_{sp,i_{2}{\left({PO}_{4}\right)}_{3}}\right)}^{\frac{1}{5}}\right)}^{2}\\ & +k_{cryst,Mg{NH}_{4}{PO}_{4}}{\left({\left(c_{{Mg}^{2+}}c_{{NH}_{4}^{+}}c_{{PO}_{4}^{3-}}\right)}^{\frac{1}{3}}-{\left(K_{sp,Mg{NH}_{4}{PO}_{4}}\right)}^{\frac{1}{3}}\right)}^{2}\\ & +k_{cryst,KMg{PO}_{4}}{\left({\left(c_{K^{+}}c_{{Mg}^{2+}}c_{{PO}_{4}^{3-}}\right)}^{\frac{1}{3}}-{\left(K_{sp,KMg{PO}_{4}}\right)}^{\frac{1}{3}}\right)}^{2},\;\;\;i\in \left\{Ca,Co,Fe,Ni\right\} \end{array}$$

(5) $$\begin{array}{ll} \rho_{l-s,i^{2+}} & =k_{cryst,{iCO}_{3}}\;{\left({\left(c_{i^{2+}}\;c_{{CO}_{3}^{2-}}\right)}^{\frac{1}{2}}-{\left(K_{sp,{iCO}_{3}}\right)}^{\frac{1}{2}}\right)}^{2}+{3\;k}_{cryst,i_{3}{\left({PO}_{4}\right)}_{2}}{\left({\left({\left(c_{i^{2+}}\right)}^{3}\;{\left(c_{{PO}_{4}^{3-}}\right)}^{2}\right)}^{\frac{1}{5}}-{\left(K_{sp,i_{3}{\left({PO}_{4}\right)}_{2}}\right)}^{\frac{1}{5}}\right)}^{2}\\ & +k_{cryst,iS}\;{\left({\left(c_{i^{2+}}\;\;c_{S^{2-}}\right)}^{\frac{1}{2}}-{\left(K_{sp,iS}\right)}^{\frac{1}{2}}\right)}^{2},\;\;\;i\in \left\{Ca,Co,Cu,Fe,K,Mg,Ni,Pb,Zn\right\} \end{array}$$

where the symbols $k_{cryst}\;\left({day}^{-1}\right)$ and $K_{sp}\;\left(g\;L^{-1}\right)$ are the crystallization rate and solubility product constants of precipitates from the set $I_{prec}$, respectively, while the symbol $c_{k}\;\left(g\;L^{-1}\right)$ denotes the concentration of the corresponding $k^{th}$ compound in ionic form (sulfide, carbonate, phosphate, and TMs).

The precipitation rates of sulfides in Equation (2) are obtained from precipitation/dissolution reactions ${\left(TMs\right)}_{\left(l\right)}^{2+}+S_{\left(l\right)}^{2-}↔{\left(TMs\right)S}_{\left(s\right)}$, where the TMs are Co, Cu, Fe, Ni, Pb, and Zn. The precipitation/dissolution reactions ${\left(TMs\right)}_{\left(l\right)}^{2+}+{CO}_{3\;\left(l\right)}^{2-}↔{\left(TMs\right)CO}_{3\;\left(s\right)}$, where the TMs are Ca, Co, Cu, Fe, Mg, Ni, Pb, and Zn, are the basis for precipitation rates of carbonates in Equation (3). Furthermore, Equation (4) is obtained from the reaction ${3\;\left(TMs\right)}_{\left(l\right)}^{2+}+{2PO}_{4\;\left(l\right)}^{3-}↔{{\left(TMs\right)}_{3}\left({PO}_{4}\right)}_{2\;\left(s\right)}$, where the TMs are Ca, Co, Fe, and Ni, and from the reactions ${Mg}_{\left(l\right)}^{2+}+{NH}_{4\;\left(l\right)}^{+}{+PO}_{4\;\left(l\right)}^{3-}↔{Mg{NH}_{4}PO}_{4\;\left(s\right)}$ and $K_{\left(l\right)}^{+}+{Mg}_{\left(l\right)}^{2+}{+PO}_{4\;\left(l\right)}^{3-}↔{KMgPO}_{4\;\left(s\right)}$. All these precipitation/dissolution reactions are used to formulate Equation (5), where the precipitation rates of ionic forms of TMs are determined. All already mentioned precipitation/dissolution reactions represent the basis for the determination of the concentrations of precipitates.

The concentrations of the precipitates, formed with ionic forms of appropriate TMs during the batch AD process, are computed as follows:

(6) $$\frac{dc_{{iCO}_{3}}}{dt}=k_{cryst,{iCO}_{3}}{\left({\left(c_{i^{2+}}c_{{CO}_{3}^{2-}}\right)}^{\frac{1}{2}}-{\left(k_{sp,{iCO}_{3}}\right)}^{\frac{1}{2}}\right)}^{2},\;\;i\in \left\{Ca,Co,Cu,Fe,Mg,Ni,Pb,Zn\right\}$$

(7) $$\frac{dc_{i_{3}{\left({PO}_{4}\right)}_{2}}}{dt}=k_{cryst,i_{3}{\left({PO}_{4}\right)}_{2}}{\left({\left({\left(c_{i^{2+}}\right)}^{3}{\left(c_{{PO}_{4}^{3-}}\right)}^{2}\right)}^{\frac{1}{5}}-{\left(k_{sp,i_{3}{\left({PO}_{4}\right)}_{2}}\right)}^{\frac{1}{5}}\right)}^{2},\;\;i\in \left\{Ca,Co,Fe,Ni\right\}$$

(8) $$\frac{dc_{iS}}{dt}=k_{cryst,iS}{\left({\left(c_{i^{2+}}c_{S^{2-}}\right)}^{\frac{1}{2}}-{\left(k_{sp,iS}\right)}^{\frac{1}{2}}\right)}^{2},\;\;i\in \left\{Co,Cu,Fe,Ni,Pb,Zn\right\}$$

The upgraded BioModel, which comprises 80 ordinary differential equations (ODEs) and 54 algebraic equations (AEs), contains 187 model parameters, which are unknown or hard to determine. These model parameters, $x_{m,i},\;i=1\ldots N_{m}\;\left(N_{m}=187\right)$, which have to be calibrated, can be summarized as follows:

- Initial influent concentrations of 13 types of bacteria in the feeding substrate, $X_{j}\;\left(g\;L^{-1}\right),\;j\in I_{bac}$ and concentrations of three types of enzymes in the feeding substrate for degrading carbohydrates, proteins, and lipids, $c_{E0,i}\left(g\;L^{-1}\right),\;i\in I_{enz}$;
- Thirteen maximal microbial grow rates at optimal temperature, $\mu_{j,max,T_{opt}}\left({day}^{-1}\right),\;j{\in I}_{bac};$ 13 microbial decays as a percentage of maximal microbial growth rates $b_{j,dec}\;\left(/\right),\;j{\in I}_{bac}$; 26 parameters (${pK}_{j}^{lo}\;\left(/\right)$, ${pK}_{j}^{up}\;\left(/\right)$,$\;j\in I_{bac}$) which are included in the pH function $f_{pH,j}$ describing the pH effects on the growth rate using the Michaelis pH function; 13 parameters $\alpha_{j}\left({℃}^{-1}\;{day}^{-1}\right),\;j\in I_{bac}$; 13 optimal temperatures $T_{j,opt}\left(℃\right);$ and 13 maximal temperatures $T_{j,max}\left(℃\right),\;j\in I_{bac}$ for the growth rate of $j^{th}$ microbial type;
- Hydrolysis rate constants $k_{hyd,i}\left({day}^{-1}\right),\;i{\in I}_{hyd}$ and three Michaelis–Menten half-saturation constants $K_{ME0,i}\;\left(g\;L^{-1}\right),\;i{\in I}_{hyd}$;
- Sixteen Monod saturation constants $k_{M}\;\left(g\;L^{-1}\right)$ related to various substrates and bacteria: $k_{M,suAsu},$ $k_{M,aaAaa},$ $k_{M,glyAgly},$ $k_{M,oaAoa},$ $k_{M,proApro},$ $k_{M,buAbu},$ $k_{M,vaAva},$ $k_{M,H_{2}Mhyd},$ $k_{M,acMac},$ $k_{M,Sio,atSs},$ $k_{M,proSpro},$ $k_{M,Sio,atSpro},$ $k_{M,acSac},$ $k_{M,Sio,atSac},$ $k_{M,H_{2}Shyd},$ and $k_{M,Sio,atShyd}$;
- Twenty-six inhibition constants $K_{inh}\left(g\;L^{-1}\right)$ related to (a) VFA inhibition of hydrolysis process $K_{inh,VFA}$ and (b) compound and metal ion inhibition of the growth of various bacteria $K_{{inh,H}_{2},Agly},$ $K_{{inh,H}_{2},Aoa},$ $K_{{inh,H}_{2},Apro},$ $K_{{inh,H}_{2}S,Apro},$ $K_{{inh,H}_{2},Abu},$ $K_{inh,H_{2}S,Abu},$ $K_{{inh,H}_{2},Ava},$ $K_{{inh,H}_{2}S,Ava},$ $K_{{inh,H}_{2}S,Mac},$ $K_{{inh,NH}_{3},Mac},$ $K_{{inh,Cu}^{2+},Abu},$ $K_{{inh,Zn}^{2+},Abu},$ $K_{{inh,Cr}^{2+},Abu},$ $K_{{inh,Pb}^{2+},Abu},$ $K_{{inh,Ni}^{2+},Abu},$ $K_{{inh,Cu}^{2+},Mac},$ $K_{{inh,Zn}^{2+},Mac},$ $K_{{inh,Cr}^{2+},Mac},$ $K_{{inh,Pb}^{2+},Mac},$ $K_{{inh,Ni}^{2+},Mac},$ $K_{inh{,H}_{2}S,Mhyd},$ $K_{inh,H_{2}S,Ss},$ $K_{{inh,H}_{2}S,Spro},$ $K_{inh,H_{2}S,Sac},$ and $K_{{inh,H}_{2}S,Shyd},$ and two limitation factors $K_{M}\left(g\;L^{-1}\right)$ of inorganic nitrogen ($K_{M,N_{io}}$) and inorganic phosphorus ($K_{M,P_{io}}$) related to all microbial growth rates;
- Ten parameters of mass transfer rates from liquid to gas phase ${\left(K_{L}a\right)}_{j,a}\left({℃}^{-1}\;{day}^{-1}\right)$ and ${\left(K_{L}a\right)}_{j,b}\left({day}^{-1}\right),\;j\in I_{gas}$;
- Twenty precipitation rate constants $k_{cryst}\;\left({day}^{-1}\right):$ $k_{{cryst,CaCO}_{3}},$ $k_{{cryst,CoCO}_{3}},$ $k_{{cryst,CuCO}_{3}},$ $k_{{cryst,FeCO}_{3}},$ $k_{{cryst,MgCO}_{3}},$ $k_{{cryst,NiCO}_{3}},$ $k_{{cryst,PbCO}_{3}},$ $k_{{cryst,ZnCO}_{3}},$ $k_{cryst,CoS},$ $k_{cryst,CuS},$ $k_{cryst,FeS},$ $k_{cryst,NiS},$ $k_{cryst,PbS},$ $k_{cryst,ZnS},$ $k_{{cryst,Ca}_{3}{\left(PO_{4}\right)}_{2}},$ $k_{{cryst,Co}_{3}{\left(PO_{4}\right)}_{2}},$ $k_{{cryst,Fe}_{3}{\left(PO_{4}\right)}_{2}},$ $k_{{cryst,Ni}_{3}{\left(PO_{4}\right)}_{2}},$ $k_{{cryst,MgNH_{4}PO}_{4}}$, and $k_{{cryst,KMgPO}_{4}}$.

The values of the 20 precipitation rate constants and 10 inhibition constants, which are directly connected to the modeling of TM activities during the AD process, as well as the other 157 model parameters, are determined using a self-developed calibration procedure.

**Sensitivity analysis.** The sensitivity analysis was performed to determine the so-called importance factor $f_{IM,i}$ of each $i^{th}$ model parameter from the set of all $N_{m}$ parameters. For this reason, random values of $5\cdot N_{m}$ AD model designs were generated (each design is a complete set of all $N_{m}$ model parameters). The importance of model parameters is determined by their effects on the ${CH}_{4}$ flow rate.

**Calibration procedure.** In order to calibrate the $N_{m}$ model parameters, the ASO procedure was used [28,29,30]. The ASO procedure incorporates the upgraded BioModel and the sensitivity analysis with a gradient-based optimization algorithm. The initial values of all model parameters (which are linearly mapped to design variables) were taken as recommended from the available literature. At the beginning of the first stage, some initial and a relatively high activation threshold value $f_{T}$ was chosen to determine a relatively low number of active design parameters. Within each stage, the active design variables $x_{m,i}^{*}$, for which it holds that the importance factor is greater than the threshold $f_{IM,i}\geq f_{T}$, are determined, while all other design variables are designated as passive in the current stage. The values of active design parameters are optimized while keeping the passive ones constant at their recommended values. After that, a new stage with a lower value of threshold $f_{T}$ is started until all design variables are active and calibrated using the optimization procedure.

**BioModel parameter calibration.** A unified optimization procedure was used to calibrate the model parameters $x_{mp,i},\;i=1\ldots N_{m}$. The design variables $x_{m,i}$ are defined as normalized model parameters by the relation $x_{m,i}=\frac{x_{mp,i}-x_{mp,i}^{LO}}{x_{mp,i}^{UP}-x_{mp,i}^{LO}}$, where $x_{mp,i}^{LO}$ and $x_{mp,i}^{UP}$ are the corresponding lower and upper limits of the $i^{th}$ model parameter $x_{mp,i}$ to be optimized, respectively. The design variables $x_{m,i}$, the objective function $g_{0}$, as well as the constraint $g_{i}$ are defined in various ways, as follows.

The objective function $g_{0,cal}$ is defined as an integral of daily deviations between the simulated and measured ${CH}_{4}$ flow rates, as shown in Equation (9). These deviations should be minimized. Furthermore, the constraints, which are defined in the standard form $g_{i,cal}\leq 0$, are related to the maximal bacteria concentration by limiting the concentration to below the maximal value of $x_{bac}^{max}$, as shown in Equation (10), and to various AD performances by limiting the lower [Equation (11)] and upper [Equation (12)] values of ${CH}_{4}$ flow rates, by limiting the minimal value of the produced ${CH}_{4}$ [Equation (13)], and by prescribing the maximal allowed fractions of the produced $H_{2},H_{2}S,andNH_{3}$ in biogas [Equations (14)–(16)]. These functions are as described as follows:

(9) $$g_{0,cal}=\psi_{0,cal}\int_{0}^{t_{total}}{\left(\frac{Q_{{CH}_{4}}\left(t\right)-Q_{{CH}_{4,exp}}\left(t\right)}{{\bar{Q}}_{{CH}_{4,exp}}}\right)}^{2}dt$$

(10) $$g_{1,cal}=\frac{\sum_{j}\left(X_{j}-X_{bac}^{max}\right)}{X_{bac}^{max}},\;\;j\in I_{bac}$$

(11) $$g_{2,cal}=\frac{\int_{0}^{t_{total}}\left(0.5+\tan^{-1}\left(\frac{10\left(k^{LO}Q_{{CH}_{4,exp}}\left(t\right)-Q_{{CH}_{4}}\left(t\right)\right)}{{\bar{Q}}_{{CH}_{4,exp}}}\right)\left(k^{LO}Q_{{CH}_{4,exp}}\left(t\right)-Q_{{CH}_{4}}\left(t\right)\right)\right)dt}{t_{total}\;\;{\bar{Q}}_{{CH}_{4,exp}}}$$

(12) $$g_{3,cal}=\frac{\int_{0}^{t_{total}}\left(0.5+\tan^{-1}\left(\frac{10\left(Q_{{CH}_{4}}\left(t\right)-k^{UP}Q_{{CH}_{4,exp}}\left(t\right)\right)}{{\bar{Q}}_{{CH}_{4,exp}}}\right)\left(Q_{{CH}_{4}}\left(t\right)-k^{UP}Q_{{CH}_{4,exp}}\left(t\right)\right)\right)dt}{t_{total}\;\;{\bar{Q}}_{{CH}_{4,exp}}}$$

(13) $$g_{4,cal}=\psi_{4,cal}\left(\frac{\left|V_{{CH}_{4}}-V_{{CH}_{4,exp}}\right|}{V_{{CH}_{4,exp}}}-\varphi_{{CH}_{4}}\right)$$

(14) $$g_{5,cal}=\psi_{5,cal}\left(\frac{V_{H_{2}}}{V_{biogas}}-\varphi_{H_{2}max}\right)$$

(15) $$g_{6,cal}=\psi_{6,cal}\left(\frac{V_{H_{2}S}}{V_{biogas}}-\varphi_{H_{2}S,max}\right)$$

(16) $$g_{7,cal}=\psi_{7,cal}\left(\frac{V_{{NH}_{3}}}{V_{biogas}}-\varphi_{{NH}_{3},max}\right)$$

where $\psi_{0,cal},\;\psi_{4,cal}$, $\psi_{5,cal}$, $\psi_{6,cal}$, and $\psi_{7,cal}$ are normalization constants; $t_{total}$ is the AD process duration; and the symbols $Q_{{CH}_{4}}\left(t\right)\;\left(L\;{day}^{-1}\right)$, $Q_{{CH}_{4},exp}\left(t\right)\;\left(L\;{day}^{-1}\right)$, and ${\bar{Q}}_{{CH}_{4,exp}}\left(L\;{day}^{-1}\right)$ denote time-dependent simulated values, time-dependent measured values, and the average values of the measured ${CH}_{4}$ flow rates, respectively. The symbol $X_{j}\;\left(g\;L^{-1}\right)$ denotes the concentration of $j^{th}$ bacteria type from the set $I_{bac}$, while $X_{bac}^{max}\;\left(g\;L^{-1}\right)$ denotes the maximal allowed initial concentration of all bacteria. The symbols $V_{{CH}_{4}}\;\left(L\right)$ and $V_{{CH}_{4},exp}\;\left(L\right)$ are the simulated and measured volumes of the produced ${CH}_{4}$, while $V_{H_{2}}$, $V_{H_{2}S},$ $V_{{NH}_{3}}$, and $V_{biogas}$ represent the simulated volumes of hydrogen, hydrogen sulfide, ammonia, and biogas, respectively. Finally, the symbol $\varphi_{{CH}_{4}}$ denotes the prescribed fraction of ${CH}_{4}$, while the symbols $\varphi_{H_{2}max}$, $\varphi_{H_{2}S,max}$, and $\varphi_{{NH}_{3},max}$ are the prescribed maximal allowed fractions of hydrogen, hydrogen sulfide, and ammonia, respectively, in the produced biogas. The inverse tangent function in Equations (11) and (12) was employed as a smooth substitute for the conventional step function. This choice is essential to maintain the differentiability of the involved functions, a requirement for using a gradient-based optimizer.

**Statistical evaluation methodology.** For the evaluation of the proposed upgraded BioModel, the measured AD performance is compared with NS using four statistical indicators (SIs): (i) mean absolute error $\varepsilon_{MAE}$, (ii) root mean square error $\varepsilon_{RMSE}$, (iii) coefficient of determination $R^{2}$, and (iv) the relative index of agreement $I_{A,rel}$ [29].

### 2.3. Optimization of the AD Process

The design variables $x_{p,i}$ were related to some process parameters $x_{pp,i},\;i=1\ldots N_{p}$, and more precisely to the concentrations of TMs (Ca,Co,Cr,Cu,Fe,K,Mg,Na,Ni, Pb,Zn) in the feeding substrate. Thus, there are a total of 11 design variables $x_{p,i},\;i=1\ldots N_{p},\;\left(N_{p}=11\right)$ that are defined as normalized process parameters based on the relation $x_{p,i}=\frac{x_{pp,i}-x_{pp,i}^{LO}}{x_{pp,i}^{UP}-x_{pp,i}^{LO}}$, where $x_{pp,i}^{LO}$ and $x_{pp,i}^{UP}$ are the corresponding lower and upper limits of the $i^{th}$ process parameter $x_{pp,i},\;i=1\ldots N_{p}$ to be optimized, respectively.

Three different optimization tasks were formulated using various definitions of objective $g_{0}$ and constraint $g_{i}$ functions; these optimization tasks are labeled as Cases A, B, and C. In Case A, a single objective function is defined, while in Cases B and C the multi-objective optimization problems are defined by combining multiple objectives into a single-objective function. This is achieved by summing individual objective functions, multiplied by adequate weighting factors.

In Case A, the produced biogas volume is maximized in the shortest production time possible, as noted in Equation (17). This is achieved using the time-integrated biogas volume, where maximizing the objective function will increase both the total biogas volume and the biogas production rate. In Case B, the produced biogas volume is maximized in the shortest production time possible, and the $H_{2}S$ production is minimized, as shown in Equation (18). The objective function, defined by Equation (19), promotes maximal ${CH}_{4}$ volume in the shortest production time possible and minimal $H_{2}S$ production. The imposed constraints in Case A are related only to the upper and lower values of the design variables $x_{p,i},\;i=1\ldots N_{p}$. In addition to these, the constraint in Case B is related to the maximal allowed $H_{2}S$ fraction in biogas, as shown in Equation (20); in Case C, the constraints are related to the maximal allowed fractions of $H_{2}S$, $H_{2}$, and $NH_{3}$ in the produced biogas, as shown in Equations (20)–(22). These objective and constraint functions are provided below:

(17) $$g_{0}=-\psi_{0}\int_{0}^{t_{total}}V_{biogas}\left(t\right)dt$$

(18) $$g_{0}=-\psi_{01}\int_{0}^{t_{total}}V_{biogas}\left(t\right)+\psi_{02}V_{H_{2}S}$$

(19) $$g_{0}=-\psi_{01}\int_{0}^{t_{total}}V_{{CH}_{4}}\left(t\right)dt+\psi_{02}V_{H_{2}S}$$

(20) $$g_{1}=\psi_{1}\left(\frac{V_{H_{2}S}}{V_{biogas}}-\varphi_{H_{2}S,max}\right)$$

(21) $$g_{2}=\psi_{2}\left(\frac{V_{H_{2}}}{V_{biogas}}-\varphi_{H_{2},max}\right)$$

(22) $$g_{3}=\psi_{3}\left(\frac{V_{NH_{3}}}{V_{biogas}}-\varphi_{NH_{3},max}\right)$$

where $\psi_{0}$, $\psi_{01}$, $\psi_{02}$, $\psi_{1}$, $\psi_{2}$, $\psi_{3}$, and $\psi_{4}$ are the weighting factors; $\varphi_{H_{2}S,max}$, $\varphi_{H_{2},max}$, and $\varphi_{NH_{3},max}$, denote maximal fractions of $H_{2}$, $H_{2}S$, and ${NH}_{3}$ in the produced biogas, respectively. The symbols $V_{biogas}\;\left(L\right)$, $V_{{CH}_{4}}\;\left(L\right)$, $V_{H_{2}}\;\left(L\right)$, $V_{H_{2}S}\;\left(L\right)$, and $V_{NH_{3}}\;\left(L\right)$ denote the volumes of biogas, ${CH}_{4}$, $H_{2}$, $H_{2}S$, and ${NH}_{3}$, respectively.

### 2.4. Solution Procedure

The proposed BioModel, the ASO procedure, and the whole optimization procedure were coded in-house in C# language. To solve the system of ODEs, the Runge–Kutta and Euler methods are used. The engaged gradient-based optimization algorithm is based on an approximation method [39,40] that sequentially generates approximate strictly convex and separable nonlinear programming problems and solves them to generate a sequence of converging approximate solutions. The algorithm uses the history of design derivatives of the objective and constraint functions to gradually improve the quality of the approximation. Consequently, the convergence is often relatively fast and stable. Since the analytical derivatives cannot be obtained easily and any derived formulas would be valid only for a particular AD model form, the numerical differentiation using simple forward differences was used in this work to get the needed design derivatives.

All numerical simulations were performed using a desktop computer with Inter i7 3.2 GHz CPU with 8 cores. The CPU time for one simulation of the AD process was approximately 1 s. The CPU time for one full optimization cycle of BioModel parameter calibration, where design derivatives computation was parallelized, is up to 1 min, while 4 s are needed for one optimization cycle of the AD process.

## 3. Results and Discussion

At first, the results of sensitivity analysis, which were used in BioModel calibration, are presented. Then the AD performance results, obtained by NS using the upgraded BioModel, are compared with experimentally obtained data in the cases of BioModel calibration (bioreactor B1) and BioModel validation (bioreactors B2 and B3) and with various perturbations of calibrated model parameters. Finally, the results of AD model optimization are presented and discussed.

### 3.1. Sensitivity Analysis

**Figure 1:**
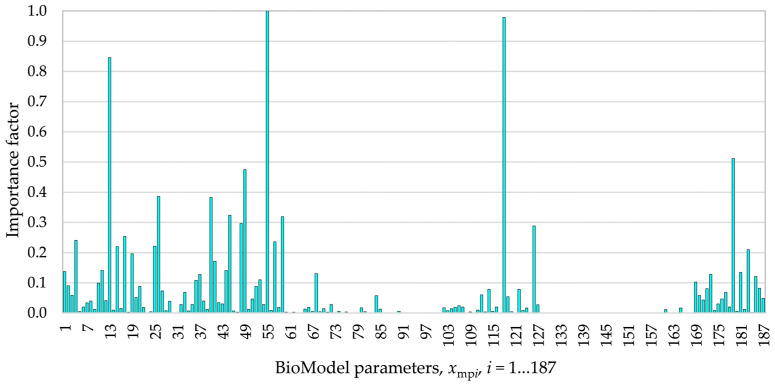
Importance factors of BioModel parameters.

The sensitivity analysis was performed for the AD process in bioreactor B1. The obtained importance factors for 187 BioModel parameters, obtained from sensitivity analysis, are presented in Figure 1.

According to Figure 1, the first ten most important BioModel parameters are the maximal growth rate of methanogenic acetate degraders at optimal temperature $\mu_{Mac,max,T_{opt}}$, lower pH drop-off value for methanogenic acetate degraders ${pK}_{Mac}^{LO}$, inhibition constant for $H_{2}S$ by methanogenic acetate degraders $K_{{inh,H}_{2}S,Mac},$ initial concentration of methanogenic acetate degraders $X_{Mac,in}$, maximal growth rate of acidogenic amino acid degraders at optimal temperature $\mu_{Aaa,max,T_{opt}}$, inhibition constant for Zn by methanogenic acetate degraders $K_{inh,Zn,Mac}$, Monod constant related to acetate and methanogenic acetate degraders $k_{M,acMac}$, Monod constant related to sulfates and sulfate-reducing bacteria $k_{M,Sio,atSac}$, maximal growth rate of sulfate-reducing bacteria of acetate at optimal temperature $\mu_{Sac,max,T_{opt}}$, and maximal growth rate of acidogenic sugar degraders at optimal temperature $\mu_{Asu,max,T_{opt}}$.

### 3.2. BioModel Calibration

The calibration of BioModel parameters was performed for the AD process in bioreactor B1. At the beginning, the mean value of interval $\left[x_{mp,i}^{LO},x_{mp,i}^{UP}\right]$ is set as the initial value (initial design) of $i^{th}$ model parameter. The lower, upper, and calibrated values of all 187 BioModel parameters are shown in Table A1 in Appendix A.

For the selection of active design variables, the threshold values of $f_{T}$ were sequentially chosen as 0.2 (Set 1), 0.1 (Set 2), 0.001 (Set 3), and 0 (Set 4, optimal design). For each set, the total number $N_{{x_{m,i}}^{*}}$ of the corresponding active design variables $x_{m,i}^{*}$, number of iterations $N_{iter}$ during the calibration, and the value of objective function $g_{0}$ are collected in Table 3. The optimized values of active design variables $x_{m,i}^{*}$, obtained by considering Set 1, were used as initial values of these variables for the optimization of the next set. The value of the objective function was gradually minimized from the initial design (ID_cal_) value of 3.1060 to the optimal design (OD_cal_, Set 4) value of 0.01185.

**Figure 2:**
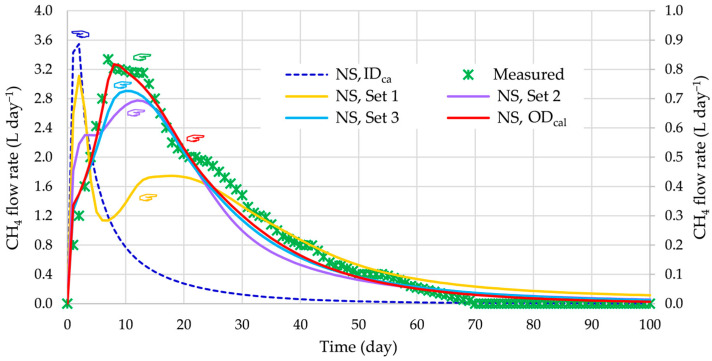
Methane flow rate during BioModel calibration using the ASO procedure.

The results of BioModel calibration by the ASO procedure are presented in Figure 2. It can be seen that the differences between measured and simulated ${CH}_{4}$ flow rates in the initial design are extremely high. Furthermore, the simulated values are becoming closer to experimental data as the number of active parameters increases. This is confirmed also by the average difference between simulated and measured ${CH}_{4}$ flow rates (average absolute daily difference divided by average measurement value). The average difference of ${CH}_{4}$ flow rates is reduced during calibration from 95.5% (initial values of model parameters; ID_cal_), through 40.9% (Set 1), 23.6% (Set 2), and 16.8% (Set 3) to 12.0% (final calibrated values of model parameters; OD_cal_). It is clearly evident that the simulated ${CH}_{4}$ flow rates, obtained under the optimal design with all calibrated BioModel parameters, agree very well with the measured values throughout the whole time period of the AD process.

**Figure 3:**
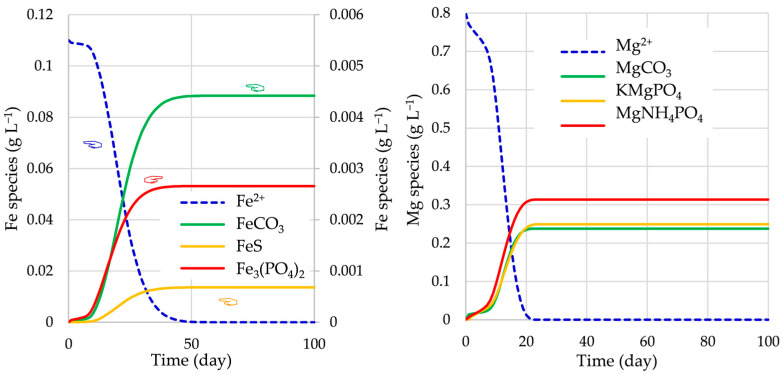
Fe and Mg species during the AD process at OD_cal_.

The upgraded BioModel enables predictions of the concentration of TEs during the AD process. As an example, the concentrations of various Fe and Mg species at OD_cal_ are presented in Figure 3.

The calibrated values of BioModel parameters are confirmed also by error, $\varepsilon_{MAE}$, and $\varepsilon_{RMSE}$ as well as efficiency, $R^{2}$ and $I_{A,rel}$, SIs, as shown in Table 4. For bioreactor B1 (BioModel calibration), the calculated values of SIs are related to methane flow rate $Q_{{CH}_{4}}$. During calibration process using the ASO procedure, from the ID_cal_ to the OD_cal_ design, the values of $\varepsilon_{MAE}$ and $\varepsilon_{RMSE}$ decrease by approximately eightfold and sixteenfold, respectively. On the other hand, the values of $R^{2}$ and $I_{A,rel}$ increase by approximately fourfold and twofold, respectively. The obtained values of all error and efficiency SIs for OD_cal_ design are acceptable.

### 3.3. BioModel Validation

**Figure 4:**
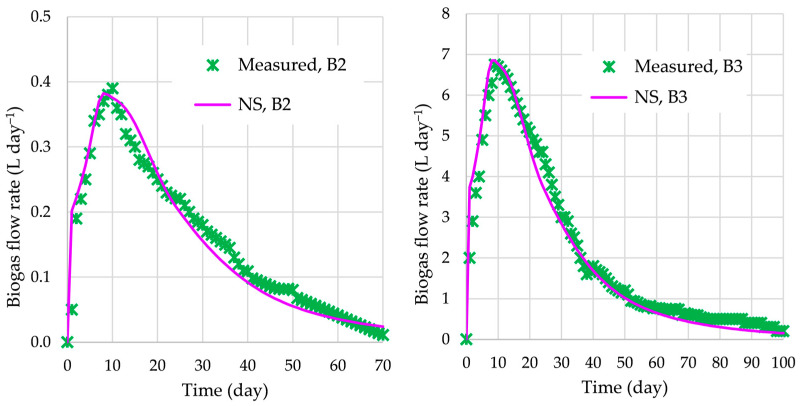
Biogas flow rates for the AD process in bioreactors B2 and B3.

The validation of BioModel parameters, which were calibrated with respect to the experimental data of the AD process in bioreactor B1, is performed for the AD processes in bioreactors B2 and B3. For these two cases, the numerically obtained biogas flow rates are compared with measured data, as shown in Figure 4. The average differences in biogas flow rates are 11.5% (bioreactor B2) and 11.2% (bioreactor B3). In both cases, the biogas flow rate obtained using NS agree very well with the experimentally obtained one. This good agreement of AD performance confirms also the efficiency of the ASO procedure for calibration.

Furthermore, with respect to the measured and simulated biogas flow rates in bioreactors B2 and B3 (BioModel validation), various SIs were calculated, as shown in Table 5. All obtained values of the error SIs are acceptable.

### 3.4. Importance of Accurate Calibration of BioModel Parameters

**Figure 5:**
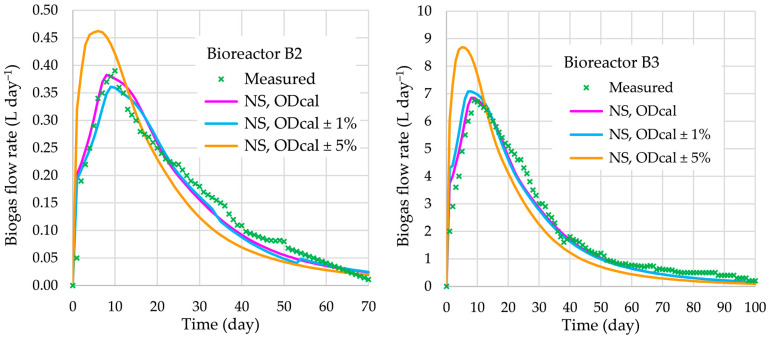
Biogas flow rates in B2 and B3; NS done using various perturbations of calibrated model parameter values.

In order to test the importance of the accuracy of calibration of all model parameters, an extra study was performed to include various perturbations of the calibrated values; the observed bioreactors are B2 and B3. For this purpose, the impact of random perturbations of all BioModel parameters on the AD performance was investigated. In the case of small perturbations, the values of model parameters were randomly generated based on a ±1% perturbation of their calibrated values (OD_cal_ ± 1%). In the case of high perturbation, the values of model parameters were randomly generated based on a ± 5% perturbation of their calibrated values (OD_cal_ ± 5%). The comparison of the simulated AD performances with the measured data are shown in Figure 5. The values of average differences in biogas flow rates in the case of validation in bioreactor B2 increases by increasing the perturbation of calibrated values of model parameters from 11.5% (OD_cal_) and 12.0% (OD_cal_ ± 1%) to 29.1% (OD_cal_ ± 5%). In the case of validation in bioreactor B3, the obtained average difference of the biogas flow rate increases from 11.2% (OD_cal_) and 14.6% (OD_cal_ ± 1%) to 34.5% (OD_cal_ ± 5%). It is evident that using 5% random perturbation of calibrated values of model parameters, the discrepancy between simulated and experimental AD performance can increase up to threefold with respect to those obtained with OD_cal_.

The comparison of biogas flow rates in Figure 5 clearly shows that the disagreement increases by increasing the perturbation of model parameters. A similar conclusion can be made also by comparing the SIs, as shown in Table 6.

Increasing perturbations of the calibrated values of model parameters results in progressively degraded accuracy of simulated performances. The values of $\varepsilon_{MAE}$ and $\varepsilon_{RMSE}$ increase, while the values of $R^{2}$ and $I_{A,rel}$ decrease. The results in Figure 5 and Table 6 show that accurate calibration of all model parameters is very important.

### 3.5. Optimized AD Process

In order to improve AD performance, the concentrations of all considered light TMs (Ca, K, Mg, and Na) and heavy TMs (Co, Cr, Cu, Fe, Ni, Pb, and Zn) are optimized. The prescribed allowed lower and upper values of TMs concentrations, actual initial TMs concentrations in the feeding substrate of bioreactor B1, as well as the optimal values of TMs in optimization Cases A, B, and C are given in Table 7. The lower values are prescribed as 10% of the initial concentration in the feeding substrate; these TM concentration values can be achieved by removing 90% of TMs from the feeding substrate. The upper values of TM concentrations can be achieved by adding TMs to the feeding substrate. As it is evident from Table 7, only the initial value of Na concentration remains the same in all optimization cases. This result is not surprising because the Na concentration in the feeding substrate in the initial design is already in the optimal range for the growth of mesophilic hydrogenotrophic methanogens and the associated methane production [19].

**Figure 6:**
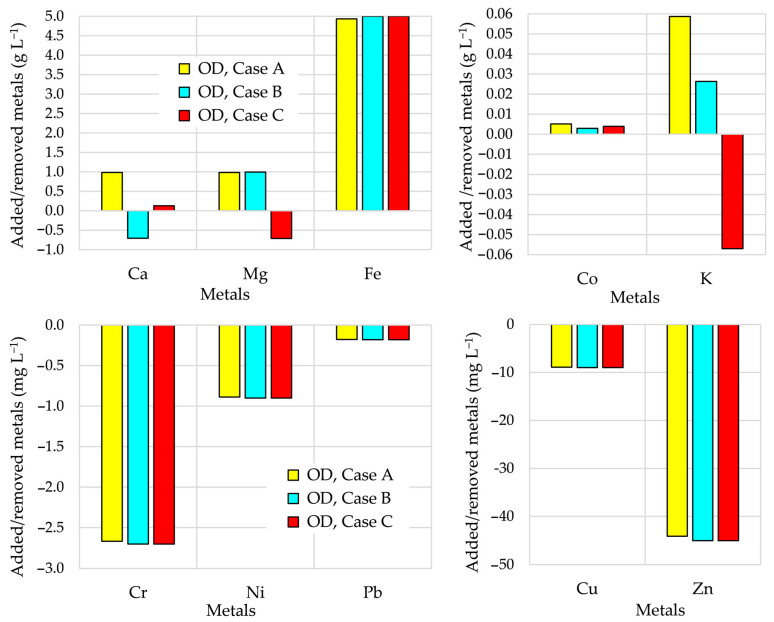
The concentrations of added/removed TMs in optimization Cases A, B, and C.

**Figure 7:**
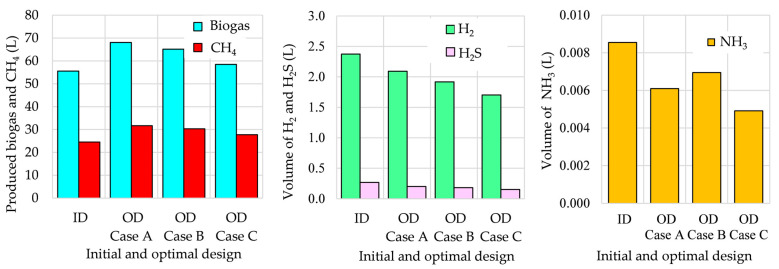
The volumes of the produced biogas, CH_4_, H_2_, H_2_S, and NH_3_ in optimization Cases A, B, and C.

**Figure 8:**
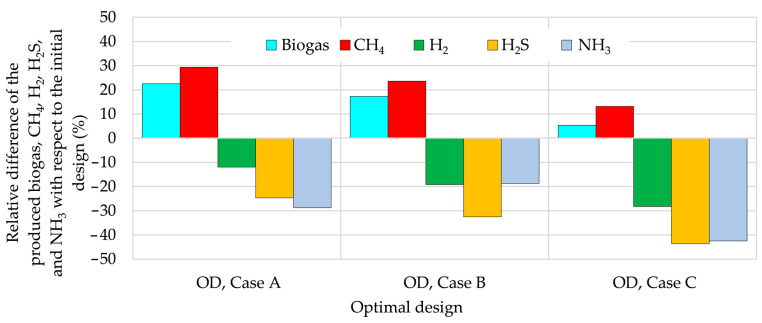
Variation in cumulative volume of biogas,$CH_{4}$, $H_{2}$, $H_{2}S$, and $NH_{3}$ with respect to initial design.

**Figure 9:**
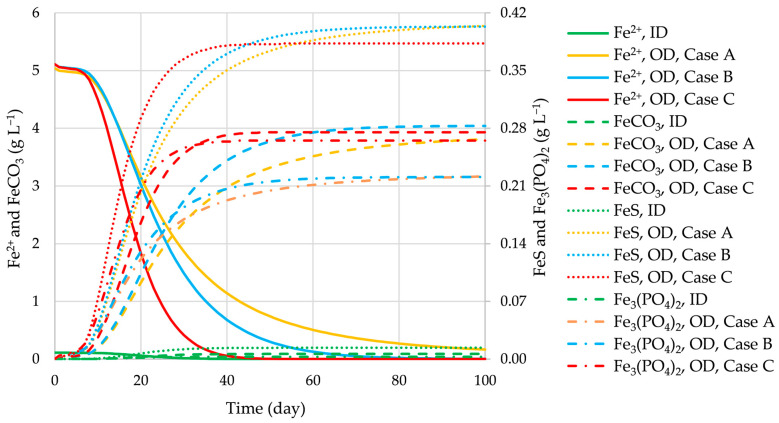
Iron species in the initial and optimal designs in Cases A, B, and C.

Some results of AD process optimization are shown in Figure 6, Figure 7, Figure 8 and Figure 9. The optimized amounts of added/removed TMs in the optimization Cases A, B, and C with respect to the initial TM concentrations in the feeding substrate are shown in Figure 6. It is evident that in all cases, the heavy metals Cr, Ni, Pb, Cu, and Zn have to be removed from the feeding substrate before starting the AD process, while Fe and Co should be added. The light TMs should be added or removed independently of the optimization case. All light TMs should be added in all cases, with the exceptions of removing Ca in Case B as well as Mg and K in Case C.

Using these optimized concentrations of TMs in the feeding substrate, the quality and quantity of the produced biogas are improved in all cases, as shown in Figure 7. The obtained results show that the highest biogas production is reached in Case A, where the objective of optimization is related only to the maximization of biogas volume. As expected, by including other requirements in the objective and constraint functions (Cases B and C), the improvement in biogas production becomes smaller. By comparing the obtained optimal designs with the initial design in Figure 8, the biogas production increases by 22.6%, 17.3%, and 5.4% in Cases A, B, and C, respectively. The CH_4_ production with respect to the initial design increases by 29.4%, 23.6%, and 13.2% in Cases A, B, and C, respectively.

By analyzing the results presented in Figure 7, it can be concluded that the biogas quality increases from Case A through Case B to Case C. For example, the produced biogas in Case C contains 7.4% more CH_4_, 31.9% less H_2_, 46.5% less H_2_S, and 45.4% less NH_3_ with respect to the initial design, as shown in Figure 8. The highest content of CH_4_ in Case C is a consequence of lower concentrations of Ca, Mg, and K in the feeding substrate. Namely, the obtained optimal Ca^2+^, Mg^2+^, and K^+^ concentrations are important for dehydrogenase activity, cell viability, and maintenance of the morphology of microbes. Higher K^+^ concentrations could lead to the displacement of exchangeable metal ions from exchangeable sites and consequently to the loss of micronutrients important for methanogens activity, or to cell dehydration, leading to the inhibition of methanogen activity [19]. Since the methanogens are less tolerant to high Mg^2+^ and Ca^2+^ concentrations, the maximization of CH_4_ production in Case C results in the lowest optimized Mg^2+^ concentration.

The main impact for the reduction of the content of H_2_S in biogas is due to the added amount of Fe^2+^ in the feeding substrate. The Fe^2+^ ions form various precipitates, such as carbonates, sulfates, and phosphates. The concentrations of various iron species during the AD process are presented in Figure 9. It is clearly evident that the formation of iron precipitates (sulfide, carbonate, and phosphate) is the fastest in the initial stage of the AD process in Case C (at optimal design) and that the amounts of FeS formed during the AD process at optimal designs in Cases A, B, and C are significantly larger than those obtained with the initial designs. Consequently, the content of H_2_S in the produced biogas is the smallest in Case C; the reduction of H_2_S with respect to initial design is 46.5%, followed by 42.5% in Case B and 38.6% in Case A, as shown in Figure 8.

An analysis of the obtained results of the AD process optimization shows the following findings: (i) the amount of H_2_S in biogas depends not only on the availability of Fe^2+^ ions but also on the formation of FeS at the initial stage of the AD process; (ii) the necessary reductions in metal concentrations in the feeding substrate, obtained during AD process optimization, require appropriate pretreatments of the feeding substrate, including adsorption of various light and heavy metals by various adsorbents (biochar, active carbon, nanomaterials, etc.) or by the addition of various substrates with lower content of metals; (iii) by maximizing the production of CH_4_ and minimizing the content of H_2_S simultaneously and by introducing the limits on the content of H_2_, H_2_S, and NH_3_ by the constraint functions (Case C), the optimal concentrations of TMs increased biogas and CH_4_ production by 5.4% and 13.5%, respectively, while H_2_, H_2_S, and NH_3_ decreased by 28.2%, 43.6%, and 42.5%, respectively. Furthermore, the content of CH_4_ increases by 7.4%, while H_2_, H_2_S, and NH_3_ decreases by 31.9%, 46.5%, and 45.4%, respectively.

## 4. Conclusions

This study deals with the modeling, calibration, validation, and numerical optimization of the AD process with special attention given to the role of TMs in batch anaerobic digestion. For this reason, a mechanistically inspired BioModel was enhanced by modeling TM activities in biochemical, chemical, and physicochemical processes; all 187 model parameters, including TM-related parameters, were calibrated by considering their interactions. The average differences in the observed AD performance in bioreactors B1, B2, and B3 are 11.6%±0.4% (calibrated model parameters, OD_cal_). A relatively small increase in average differences in biogas flow rate in bioreactors B2 and B3 is obtained at low perturbation of calibrated parameters. Meanwhile, at high perturbation (OD_cal_ ± 5%), the average differences increase up to 34.5%; a random ±5% perturbation of calibrated values of model parameters results in rather inaccurate predictions of AD performance. Finally, by optimizing TM concentrations in the feeding substrate, the quantity and quality of the produced biogas can be improved essentially; the biogas and CH_4_ production increased by 5.4% and 13.5%, respectively, while H_2_, H_2_S, and NH_3_ decreased by 28.2%, 43.6%, and 42.5%, respectively. In the future, further research focusing on the effects of multiple metals in bioreactors on the AD process as well as on the specific thresholds of the stimulatory and inhibitory concentrations during various stages of the AD process would be highly useful.

## Figures and Tables

**Table 1:** Bioreactor data.

	Bioreactor		
Parameter	B1	B2	B3
pH value, pH (/)	8.00	8.00	7.4
Temperature, T (℃)	35.00	37.00	38.5
$\mathrm{Liquid}\;\mathrm{volume}\;\mathrm{of}\;\mathrm{bioreactor},\;V_{liq}(L)$	1.00	0.25	4.5
$\mathrm{Gas}\;\mathrm{volume}\;\mathrm{of}\;\mathrm{bioreactor},\;V_{g}(L)\;$	0.10	0.20	3.5
$\mathrm{Total}\;\mathrm{pressure}\;\mathrm{in}\;\mathrm{bioreactor},\;p_{total}(bar)$	1.0059	1.0059	1.0073

**Table 2:** Input data of feedstock composition.

Parameter	Value
$\mathrm{Carbohydrate}\;\mathrm{concentration},\;c_{ch}\;({g\;L}^{-1})$	15.0000
$\mathrm{Protein}\;\mathrm{concentration},\;c_{pr}\;({g\;L}^{-1})$	35.0000
$\mathrm{Lipid}\;\mathrm{concentration},\;c_{li}\;({g\;L}^{-1})$	3.0000
$\mathrm{Inert}\;\mathrm{matter}\;\mathrm{concentration},\;c_{inert}\;({g\;L}^{-1})$	50.0000
$\mathrm{Inorganic}\;\mathrm{carbon}\;\mathrm{concentration},\;c_{Cio}\;({g\;L}^{-1})$	2.5000
$\mathrm{Inorganic}\;\mathrm{nitrogen}\;\mathrm{concentration},\;c_{Nio}\;({g\;L}^{-1})$	2.0000
$\mathrm{Inorganic}\;\mathrm{phosphorous}\;\mathrm{concentration},\;c_{Pio}\;({g\;L}^{-1})$	2.6000
$\mathrm{Inorganic}\;\mathrm{sulfur}\;(\mathrm{sulfide})\;\mathrm{concentration},\;c_{Sioid}\;({g\;L}^{-1})$	0.1000
$\mathrm{Inorganic}\;\mathrm{sulfur}\;(\mathrm{sulfate})\;\mathrm{concentration},\;c_{Sioat}\;({g\;L}^{-1})$	0.5000
$\mathrm{Nitrogen}\;\mathrm{dioxide}\;\mathrm{concentration},\;c_{{NO}_{2}}\;({g\;L}^{-1})$	0.0080
$\mathrm{Calcium}\;\mathrm{concentration},\;c_{Ca}\;\;({g\;L}^{-1})$	3.0000
$\mathrm{Cobalt}\;\mathrm{concentration},\;c_{Co}\;({g\;L}^{-1})$	0.0200
$\mathrm{Chromium}\;\mathrm{concentration},\;c_{Cr}\;({g\;L}^{-1})$	0.0030
$\mathrm{Copper}\;\mathrm{concentration},\;c_{Cu}\;({g\;L}^{-1})$	0.0100
$\mathrm{Iron}\;\mathrm{concentration},\;c_{Fe}\;({g\;L}^{-1})$	0.1100
$\mathrm{Potassium}\;\mathrm{concentration},\;c_{K}\;({g\;L}^{-1})$	3.2000
$\mathrm{Magnesium}\;\mathrm{concentration},\;c_{Mg}\;({g\;L}^{-1})$	0.8000
$\mathrm{Sodium}\;\mathrm{concentration},\;c_{Na}\;({g\;L}^{-1})$	0.3000
$\mathrm{Nickel}\;\mathrm{concentration},\;c_{Ni}\;({g\;L}^{-1})$	0.0010
$\mathrm{Lead}\;\mathrm{concentration},\;c_{Pb}\;({g\;L}^{-1})$	0.0002
$\mathrm{Zinc}\;\mathrm{concentration},\;c_{Zn}\;({g\;L}^{-1})$	0.0500

**Table 3:** Active sets data related to the ASO procedure.

Set	$f_{T}$	$N_{{x_{m,i}}^{*}}$	$N_{iter}$	$g_{0}$
1	0.2	17	16	0.25700
2	0.1	30	59	0.05285
3	0.001	89	78	0.02280
4 (OD_cal_)	0	187	190	0.01185

**Table 4:** Statistical indicators of CH_4_ flow rate; bioreactor B1, BioModel calibration.

SIs	BioModel Calibration, Bioreactor B1				
	ID_cal_	Set 1	Set 2	Set 3	OD_cal_
$\varepsilon_{MAE}\;(L\;{day}^{-1})$	0.0583	0.0250	0.0144	0.0102	0.0074
$\varepsilon_{RMSE}\;(L\;{day}^{-1})$	0.2933	0.08438	0.0382	0.0252	0.0181
$R^{2}\;(/)$	0.2374	0.6491	0.9360	0.9810	0.9855
$I_{A,rel}\;(/)$	0.4872	0.8440	0.9774	0.9902	0.9953

**Table 5:** Statistical indicators of biogas flow rate; bioreactors B2 and B3, BioModel validation.

SIs	B2	B3
$\varepsilon_{MAE}\;(L\;{day}^{-1})$	0.0033	0.0654
$\varepsilon_{RMSE}\;(L\;{day}^{-1})$	0.0111	0.1817
$R^{2}\;(/)$	0.9780	0.9775
$I_{A,rel}\;(/)$	0.9877	0.9931

**Table 6:** Statistical indicators of biogas flow rate; perturbation of model parameters, bioreactors B2 and B3, validation period.

**Bioreactor**	Design	SIs			
		$\varepsilon_{MAE}\;(L\;{day}^{-1})$	$\varepsilon_{RMSE}\;(L\;{day}^{-1})$	$R^{2}\;(/)$	$I_{A,rel}\;(/)$
B2	OD_cal_	0.0033	0.0111	0.9780	0.9877
	OD_cal_ ± 1%	0.0036	0.0113	0.9712	0.9866
	OD_cal_ ± 5%	0.0087	0.0303	0.8681	0.9261
B3	OD_cal_	0.0654	0.1817	0.9775	0.9931
	OD_cal_ ± 1%	0.0850	0.2544	0.9571	0.9869
	OD_cal_ ± 5%	0.2010	0.6241	0.8090	0.9311

**Table 7:** TM concentration; lower, upper, initial, and optimized values.

No.	TM	$x_{pp,i}^{LO}$	$x_{pp,i}^{UP}$	ID	OD Case A	OD Case B	OD Case C
1	$c_{Ca}\;(g\;L^{-1})$	0.30000	4.00000	3.00000	3.9870	2.2957	3.1290
2	$c_{K}\;(g\;L^{-1})$	0.32000	4.20000	3.20000	3.2587	3.2263	3.1430
3	$c_{Mg}\;(g\;L^{-1})$	0.08000	1.80000	0.80000	1.7868	1.7970	0.0887
4	$c_{Na}\;(g\;L^{-1})$	0.03000	0.80000	0.30000	0.3000	0.3000	0.3000
5	$c_{Fe}\;(g\;L^{-1})$	0.01100	5.11000	0.11000	5.0431	5.1100	5.1099
6	$c_{Co}\;(g\;L^{-1})$	0.00200	0.12000	0.02000	0.0251	0.0229	0.0239
7	$c_{Cr}\;(mg\;L^{-1})$	0.30000	103.000	3.00000	0.3342	0.3000	0.3000
8	$c_{Ni}\;(mg\;L^{-1})$	0.10000	101.000	1.00000	0.1113	0.1000	0.1000
9	$c_{Pb}\;(mg\;L^{-1})$	0.02000	100.200	0.20000	0.0218	0.0200	0.0200
10	$c_{Cu}\;(mg\;L^{-1})$	1.00000	110.000	10.00000	1.1192	1.0001	1.0001
11	$c_{Zn}\;(mg\;L^{-1})$	5.00000	150.000	50.00000	5.9228	5.0003	5.0010

## Data Availability

The data presented in this study are available upon request from the corresponding author.

## Appendix A

**Table A1:** Lower, upper, and optimal (calibrated) values of BioModel parameters.

i	Parameter	$x_{mp,i}^{LO}$	$x_{mp,i}^{UP}$	Optimal Value	i	Parameter	$x_{mp,i}^{LO}$	$x_{mp,i}^{UP}$	Optimal Value
1	$k_{hyd,ch}\;({day}^{-1})$	1	10	5.28848	95	$k_{cryst,PbS}\;({day}^{-1})$	10	100	55.0005
2	$k_{hyd,pr}\;({day}^{-1})$	1	10	6.07000	96	$k_{cryst,ZnS}\;({day}^{-1})$	10	100	54.9998
3	$k_{hyd,li}\;({day}^{-1})$	1	10	6.53405	97	$k_{cryst,CoS}\;({day}^{-1})$	500	1000	750.386
4	$K_{inh,VFA}\;(g\;L^{-1})$	0.1	0.6	0.27516	98	$k_{{cryst,Co}_{3}{\left(PO_{4}4\right)}_{2}}\;({day}^{-1})$	50	100	74.9785
5	$K_{inh,H_{2},Agly}\;(gL^{-1})$	0.01	0.9	0.47075	99	$k_{{cryst,Ca}_{3}{\left(PO_{4}4\right)}_{2}}\;({day}^{-1})$	50	100	59.3824
6	$K_{inh,H_{2},Aoa}\;(g\;L^{-1})$	0.01	0.9	0.52991	100	$k_{{cryst,Fe}_{3}{\left(PO_{4}4\right)}_{2}}\;({day}^{-1})$	50	100	74.3711
7	$K_{inh,H_{2},Apro}\;(g\;L^{-1})$	0.01	0.9	0.56099	101	$k_{{cryst,Ni}_{3}{\left(PO_{4}4\right)}_{2}}\;({day}^{-1})$	50	100	74.9990
8	$K_{inh,H_{2},Abu}\;(g\;L^{-1})$	0.01	0.9	0.41827	102	$k_{{cryst,MgNH_{4}PO}_{4}}\;({day}^{-1})$	50	200	76.4582
9	$K_{inh,H_{2},Ava}\;(g\;L^{-1})$	0.01	0.9	0.44331	103	$k_{{cryst,KMgPO}_{4}}\;({day}^{-1})$	50	200	124.992
10	$K_{inh,H_{2}S,Apro}\;(g\;L^{-1})$	0.01	0.9	0.51747	104	${pK}_{Asu}^{lo}\;(/)$	4.5	5.5	4.98825
11	$K_{inh,H_{2}S,Abu}\;(g\;L^{-1})$	0.01	0.9	0.12073	105	${pK}_{Asu}^{up}\;(/)$	7.5	8.5	8.27958
12	$K_{inh,H_{2}S,Ava}\;(g\;L^{-1})$	0.01	0.9	0.09301	106	${pK}_{Aaa}^{lo}\;(/)$	4.5	5.5	4.98971
13	$K_{inh,H_{2}S,Mac}\;(g\;L^{-1})$	0.01	0.9	0.07857	107	${pK}_{Aaa}^{up}\;(/)$	7.5	8.5	8.48995
14	$K_{inh,H_{2}S,Mhyd}\;(g\;L^{-1})$	0.01	0.9	0.07034	108	${pK}_{Agly}^{lo}\;(/)$	4.5	5.5	5.02014
15	$K_{inh,H_{2}S,Ss}\;(g\;L^{-1})$	0.01	0.9	0.47785	109	${pK}_{Agly}^{up}\;(/)$	7.5	8.5	8.22301
16	$K_{inh,H_{2}S,Spro}\;(g\;L^{-1})$	0.01	0.9	0.25675	110	${pK}_{Aoa}^{lo}\;(/)$	4.5	5.5	5.04878
17	$K_{inh,H_{2}S,Sac}\;(g\;L^{-1})$	0.01	0.9	0.31965	111	${pK}_{Aoa}^{up}\;(/)$	7.5	8.5	8.45347
18	$K_{inh,H_{2}S,Shyd}\;(g\;L^{-1})$	0.01	0.9	0.53034	112	${pK}_{Apro}^{lo}\;(/)$	5.5	6.5	6.40484
19	$K_{inh,NH_{3},Mac}\;(g\;L^{-1})$	0.01	0.3	0.58207	113	${pK}_{Apro}^{up}\;(/)$	8.0	9.0	8.20391
20	$K_{inh,{Cu}^{2+},Abu}\;(g\;L^{-1})$	0.01	0.9	0.41420	114	${pK}_{Abu}^{lo}\;(/)$	5.5	6.5	6.31587
21	$K_{inh,{Zn}^{2+},Abu}\;(g\;L^{-1})$	0.01	0.9	0.35448	115	${pK}_{Abu}^{up}\;(/)$	8.0	9.0	8.61508
22	$K_{inh,{Cr}^{2+},Abu}\;(g\;L^{-1})$	0.01	0.9	0.44256	116	${pK}_{Ava}^{lo}\;(/)$	5.5	6.5	6.13055
23	$K_{inh,{Pb}^{2+},Abu}\;(g\;L^{-1})$	0.01	0.9	0.45450	117	${pK}_{Ava}^{up}\;(/)$	8.0	9.0	8.53030
24	$K_{inh,{Ni}^{2+},Abu}\;(g\;L^{-1})$	0.01	0.9	0.45174	118	${pK}_{Mac}^{lo}\;(/)$	5.5	6.5	6.00441
25	$K_{inh,{Cu}^{2+},Mac}\;(g\;L^{-1})$	0.01	0.9	0.51131	119	${pK}_{Mac}^{up}\;(/)$	8.0	9.0	8.34907
26	$K_{inh,{Zn}^{2+},Mac}\;(g\;L^{-1})$	0.01	0.9	0.58076	120	${pK}_{Mhyd}^{lo}\;(/)$	5.5	6.5	6.04491
27	$K_{inh,{Cr}^{2+},Mac}\;(g\;L^{-1})$	0.01	0.9	0.43723	121	${pK}_{Mhyd}^{up}\;(/)$	8.0	9.0	8.48158
28	$K_{inh,{Pb}^{2+},Mac}\;(g\;L^{-1})$	0.01	0.9	0.45489	122	${pK}_{Ss}^{lo}\;(/)$	5.5	6.5	5.96875
29	$K_{inh,{Ni}^{2+},Mac}\;(g\;L^{-1})$	0.01	0.9	0.49740	123	${pK}_{Ss}^{up}\;(/)$	7.5	8.5	8.07330
30	$K_{M,N_{io}}\;(g\;L^{-1})$	0.001	0.01	0.00513	124	${pK}_{Spro}^{lo}\;(/)$	5.5	6.5	6.21593
31	$K_{M,P_{io}}\;(g\;L^{-1})$	0.001	0.01	0.00536	125	${pK}_{Spro}^{up}\;(/)$	7.5	8.5	8.00344
32	$k_{M,suAsu}\;(g\;L^{-1})$	0.01	0.9	0.45069	126	${pK}_{Sac}^{lo}\;(/)$	5.5	6.5	5.64308
33	$k_{M,aaAaa}\;(g\;L^{-1})$	0.01	0.9	0.38091	127	${pK}_{Sac}^{up}\;(/)$	7.5	8.5	7.64437
34	$k_{M,glyAgly}\;(g\;L^{-1})$	0.01	0.9	0.28036	128	${pK}_{Shyd}^{lo}\;(/)$	5.5	6.5	6.02774
35	$k_{M,oaAoa}\;(g\;L^{-1})$	0.01	0.9	0.02559	129	${pK}_{Shyd}^{up}\;(/)$	7.5	8.5	7.98378
36	$k_{M,proApro}\;(g\;L^{-1})$	0.01	0.9	0.72705	130	$\alpha_{Asu}\;(K^{-1}\;{day}^{-1})$	0.00015	0.00019	0.00055
37	$k_{M,buAbu}\;(g\;L^{-1})$	0.01	0.9	0.86578	131	$\alpha_{Aaa}\;(K^{-1}\;{day}^{-1})$	0.00015	0.00019	0.00055
38	$k_{M,vaAva}\;(g\;L^{-1})$	0.01	0.9	0.65487	132	$\alpha_{Agly}\;(K^{-1}\;{day}^{-1})$	0.00015	0.00019	0.00055
39	$k_{M,H_{2}Mhyd}\;(g\;L^{-1})$	0.01	0.9	0.89994	133	$\alpha_{Aoa}\;(K^{-1}\;{day}^{-1})$	0.00015	0.00019	0.00055
40	$k_{M,acMac}\;(g\;L^{-1})$	0.01	0.9	0.70222	134	$\alpha_{Apro}\;(K^{-1}\;{day}^{-1})$	0.00015	0.00019	0.00055
41	$k_{M,Sio,atSs}\;(g\;L^{-1})$	0.01	0.9	0.09066	135	$\alpha_{Abu}\;(K^{-1}\;{day}^{-1})$	0.00016	0.00020	0.00055
42	$k_{M,proSpro}\;(g\;L^{-1})$	0.01	0.9	0.58511	136	$\alpha_{Ava}\;(K^{-1}\;{day}^{-1})$	0.00016	0.00020	0.00055
43	$k_{M,Sio,atSpro}\;(g\;L^{-1})$	0.01	0.9	0.61723	137	$\alpha_{Mac}\;(K^{-1}\;{day}^{-1})$	0.00015	0.00019	0.00054
44	$k_{M,acSac}\;(g\;L^{-1})$	0.01	0.9	0.24452	138	$\alpha_{Mhyd}\;(K^{-1}\;{day}^{-1})$	0.00015	0.00019	0.00055
45	$k_{M,Sio,atSac}$(g L^−1^)	0.01	0.9	0.24192	139	$\alpha_{Ss}\;(K^{-1}\;{day}^{-1})$	0.00016	0.00020	0.00055
46	$k_{M,H_{2}Shyd}\;(g\;L^{-1})$	0.01	0.9	0.33351	140	$\alpha_{Spro}\;(K^{-1}\;{day}^{-1})$	0.00016	0.00020	0.00055
47	$k_{M,Sio,atShyd}\;(g\;L^{-1})$	0.01	0.9	0.34444	141	$\alpha_{Sac}\;(K^{-1}\;{day}^{-1})$	0.00016	0.00020	0.00055
48	$\mu_{Asu,max,T_{opt}}\;({day}^{-1})$	1	10	7.27177	142	$\alpha_{Shyd}\;(K^{-1}\;{day}^{-1})$	0.00016	0.00020	0.00055
49	$\mu_{Aaa,max,T_{opt}}\;({day}^{-1})$	1	10	9.99998	143	$T_{Asu,opt}\;(℃)$	50	60	54.9854
50	$\mu_{Agly,max,T_{opt}}\;({day}^{-1})$	1	10	9.18755	144	$T_{Asu,max}\;(℃)$	60	70	65.0000
51	$\mu_{Aoa,max,T_{opt}}\;({day}^{-1})$	1	10	9.91931	145	$T_{Asa,opt}\;(℃)$	50	60	54.9685
52	$\mu_{Apro,max,T_{opt}}\;({day}^{-1})$	1	10	3.51354	146	$T_{Aaa,max}\;(℃)$	60	70	65.0000
53	$\mu_{Abu,max,T_{opt}}\;({day}^{-1})$	1	10	1.62130	147	$T_{Agly,opt}\;(℃)$	50	60	54.9975
54	$\mu_{Ava,max,T_{opt}}\;({day}^{-1})$	1	10	2.94090	148	$T_{Agly,max}\;(℃)$	60	70	65.0000
55	$\mu_{Mac,max,T_{opt}}\;({day}^{-1})$	1	10	5.46362	149	$T_{Aoa,opt}\;(℃)$	50	60	54.9941
56	$\mu_{Mhyd,max,T_{opt}}\;({day}^{-1})$	1	10	3.64307	150	$T_{Aoa,max}\;(℃)$	60	70	65.0000
57	$\mu_{Ss,max,T_{opt}}\;({day}^{-1})$	1	10	5.63776	151	$T_{Apro,opt}\;(℃)$	50	60	54.9866
58	$\mu_{Spro,max,T_{opt}}\;({day}^{-1})$	1	10	1.17762	152	$T_{Apro,max}\;(℃)$	60	70	65.0000
59	$\mu_{Sac,max,T_{opt}}\;({day}^{-1})$	1	10	9.34170	153	$T_{Abu,opt}\;(℃)$	55	65	60.0039
60	$\mu_{Shyd,max,T_{opt}}\;({day}^{-1})$	1	10	6.70977	154	$T_{Abu,max}\;(℃)$	65	75	70.0000
61	$b_{dec,Asu}\;(/)$	0.01	0.05	0.02996	155	$T_{Ava,opt}\;(℃)$	55	65	60.0130
62	$b_{dec,Aaa}\;(/)$	0.01	0.05	0.02684	156	$T_{Ava,max}\;(℃)$	65	75	70.0000
63	$b_{dec,Agly}\;(/)$	0.01	0.05	0.02985	157	$T_{Mac,opt}\;(℃)$	50	60	54.9936
64	$b_{dec,Aoa}\;(/)$	0.01	0.05	0.03009	158	$T_{Mac,max}\;(℃)$	60	70	65.0000
65	$b_{dec,Apro}\;(/)$	0.01	0.05	0.01590	159	$T_{Mhyd,opt}\;(℃)$	50	60	55.0023
66	$b_{dec,Abu}\;(/)$	0.01	0.05	0.04289	160	$T_{Mhyd,max}\;(℃)$	60	70	65.0000
67	$b_{dec,Ava}\;(/)$	0.01	0.05	0.02859	161	$T_{Ss,opt}\;(℃)$	30	40	35.1549
68	$b_{dec,Mac}\;(/)$	0.01	0.05	0.01980	162	$T_{Ss,max}\;(℃)$	60	70	65.0022
69	$b_{dec,Mhyd}\;(/)$	0.01	0.05	0.04300	163	$T_{Spro,opt}\;(℃)$	30	40	35.0021
70	$b_{dec,Ss}\;(/)$	0.01	0.05	0.03071	164	$T_{Spro,max}$(°C)	60	70	65.0000
71	$b_{dec,Spro}\;(/)$	0.01	0.05	0.03000	165	$T_{Sac,opt}\;(℃)$	30	40	36.2293
72	$b_{dec,Sac}\;(/)$	0.01	0.05	0.02867	166	$T_{Sac,max}\;(℃)$	60	70	65.0008
73	$b_{dec,Shyd}\;(/)$	0.01	0.05	0.02999	167	$T_{Shyd,opt}\;(℃)$	30	40	34.9999
74	${\left(K_{L}a\right)}_{{CO}_{2},a}\;({℃}^{-1}\;{day}^{-1})$	1	5	3.59248	168	$T_{Shyd,max}\;(℃)$	60	70	64.9999
75	${\left(K_{L}a\right)}_{{CO}_{2},b}\;({day}^{-1})$	10	20	14.97329	169	$K_{ME0,ch}\;\left(g\;L^{-1}\right)$	0.00001	0.001	0.00009
76	${\left(K_{L}a\right)}_{{CH}_{4},a}\;({℃}^{-1}\;{day}^{-1})$	1	5	2.97810	170	$K_{ME0,pr}\;\left(g\;L^{-1}\right)$	0.00001	0.001	0.00024
77	${\left(K_{L}a\right)}_{{CH}_{4},b}\;({day}^{-1})$	10	20	14.99536	171	$K_{ME0,li}\;\left(g\;L^{-1}\right)$	0.00001	0.001	0.00053
78	${\left(K_{L}a\right)}_{H_{2},a}\;({℃}^{-1}\;{day}^{-1})$	0.0001	0.001	0.00086	172	$X_{Asu}\;\left(g\;L^{-1}\right)$	0.1	0.5	0.26646
79	${\left(K_{L}a\right)}_{H_{2},b}\;({day}^{-1})$	0.001	0.01	0.00641	173	$X_{Aaa}\;\left(g\;L^{-1}\right)$	0.1	0.5	0.49999
80	${\left(K_{L}a\right)}_{H_{2}S,a}\;({℃}^{-1}\;{day}^{-1})$	0.0001	0.001	0.00053	174	$X_{Agly}\;\left(g\;L^{-1}\right)$	0.1	0.5	0.35606
81	${\left(K_{L}a\right)}_{H_{2}S,b}\;({day}^{-1})$	0.001	0.01	0.00565	175	$X_{Aoa}\;\left(g\;L^{-1}\right)$	0.1	0.5	0.48358
82	${\left(K_{L}a\right)}_{{NH}_{3},a}\;({℃}^{-1}\;{day}^{-1})$	0.0001	0.001	0.00055	176	$X_{Apro}\;\left(g\;L^{-1}\right)$	0.1	0.5	0.10110
83	${\left(K_{L}a\right)}_{{NH}_{3},b}\;({day}^{-1})$	0.001	0.01	0.00550	177	$X_{Abu}\;\left(g\;L^{-1}\right)$	0.1	0.5	0.20184
84	$k_{{cryst,CaCO}_{3}}\;({day}^{-1})$	1	10	2.26219	178	$X_{Ava}\;\left(g\;L^{-1}\right)$	0.1	0.5	0.14735
85	$k_{{cryst,MgCO}_{3}}\;({day}^{-1})$	1	10	6.23296	179	$X_{Mac}\;\left(g\;L^{-1}\right)$	0.1	0.5	0.10001
86	$k_{{cryst,FeCO}_{3}}\;({day}^{-1})$	1	10	2.45088	180	$X_{Mhyd}\;\left(g\;L^{-1}\right)$	0.1	0.5	0.31548
87	$k_{{cryst,NiCO}_{3}}\;({day}^{-1})$	1	10	5.34170	181	$X_{Spro}\;\left(g\;L^{-1}\right)$	0.1	0.5	0.25498
88	$k_{{cryst,CuCO}_{3}}\;({day}^{-1})$	1	10	2.21574	182	$X_{Sac}\;\left(g\;L^{-1}\right)$	0.1	0.5	0.10000
89	$k_{{cryst,PbCO}_{3}}\;({day}^{-1})$	1	10	5.47297	183	$X_{Ss}\;\left(g\;L^{-1}\right)$	0.1	0.5	0.41458
90	$k_{{cryst,ZnCO}_{3}}\;({day}^{-1})$	1	10	2.61502	184	$X_{Shyd}\;\left(g\;L^{-1}\right)$	0.1	0.5	0.34090
91	$k_{{cryst,CoCO}_{3}}\;({day}^{-1})$	1	10	4.96625	185	$c_{E0,ch}\;\left(g\;L^{-1}\right)$	0.00001	0.001	0.00063
92	$k_{cryst,FeS}\;({day}^{-1})$	1000	5000	2999.88	186	$c_{E0,pr}\;\left(g\;L^{-1}\right)$	0.00001	0.001	0.00046
93	$k_{cryst,NiS}\;({day}^{-1})$	500	1000	2999.88	187	$c_{E0,li}\;\left(g\;L^{-1}\right)$	0.00001	0.001	0.00054
94	$k_{cryst,CuS}\;({day}^{-1})$	500	1000	749.971					
